# Beetles that live with ants (Coleoptera, Carabidae, Pseudomorphini): A remarkable new genus and species from Guyane (French Guiana), *Guyanemorpha spectabilis* gen. n., sp. n.

**DOI:** 10.3897/zookeys.358.6298

**Published:** 2013-12-03

**Authors:** Terry L. Erwin

**Affiliations:** 1 Hyper-diversity Group, Department of Entomology, MRC-187, National Museum of Natural History, Smithsonian Institution, Washington, P.O. Box 37012, DC 20013-7012, USA

**Keywords:** False-form beetles, identification key, distribution, male genitalia, female ovipositor, Hymenoptera: Formicidae

## Abstract

Among the extensive collections currently being made in Guyane (French Guiana), adults of a large and colorful species of pseudomorphine were encountered. The adults present, for the first time in the Western Hemisphere, elytra with a marked color pattern, and in addition a size considerably beyond that of the rest of the members of all other known genera in the Western Hemisphere. Both of these attributes, however, are well known in the Australian pseudomorphine fauna. This new species is described and illustrated and a revised key to the Western Hemisphere genera is included. The type locality of *Guyanemorpha spectabilis* **gen. n.**, **sp. n.** is Guyane,Risquetout, PK20, 4.916°N, 52.516°W, 12m altitude.

## Abstract (es)

Entre las colecciones extensivas que actualmente se están conduciendo en Guyane (Guyana Francesa), los adultos de las especies grandes y coloridas de pseudomorfinos fueron encontrados. Los adultos presentan, por primera vez en el hemisferio occidental, élitros con un patrón colorido marcado, además de un tamaño mucho mayor que el resto de los miembros de todos los otros géneros en el hemisferio occidental. Ambos atributos, sin embargo, se conocen bastante bien en la fauna pseudomorfina de Australia. Esta nueva especie se describe e ilustra y una clave revisada para los géneros del hemisferio occidental se incluye. La localidad tipo de *Guyanemorpha spectabilis* **gen. n.**, **sp. n.** es Guyane, Risquetout, PK20, 4.916°N, 52.516°W, altitud de 12m.

## Introduction

**Figure 1.**
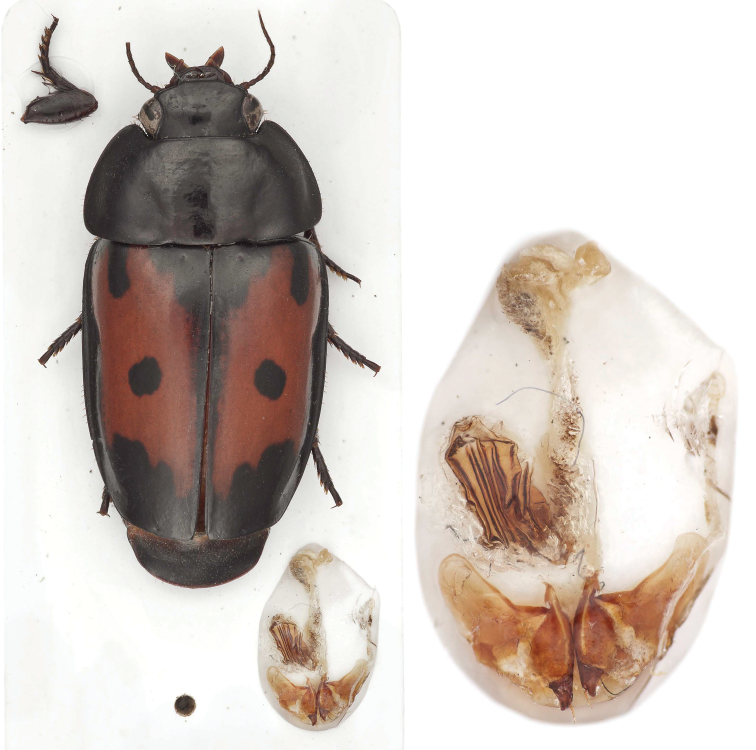
*Guyanemorpha spectabilis* sp. n., female holotype, ADP132101; Risquetout, PK20, Guyane. Habitus and female genital tract glued to card, dorsal aspect, ABL = 13.2mm.

Surprising taxa of Carabidae continue to surface as collections from remote places and new habitats are explored (e.g., Erwin 2000, 2004; Erwin and Geraci 2008). Another such taxon has been discovered in various parts of Guyane during ongoing biotic inventories in reserved areas of Guyane and exploration of that country’s insect biodiversity by The Entomological Society Antilles-Guyane (SEAG) (cf. Erwin et al. 2012). The species is remarkable because the adults (Fig. 1) present, for the first time in the Western Hemisphere, elytra with a marked color pattern, and in addition a size considerably beyond that of the rest of members of the other known genera in the Western Hemisphere. Both of these attributes, however, are well known in the Australian pseudomorphine fauna (Baehr 1992, 1997) and it is likely a greater variety of color forms will be found in the future in South America.

## Specimens and methods

Included in this study are a total of 4 specimens from other institutions and private collections (Appendix 1). “Methods and species concepts follow those previously described (Ball 1959; Erwin and Kavanaugh 1981; Kavanaugh and Erwin 1991). The species validation and diagnosis format follows as closely as possible that suggested in Erwin and Johnson (2000). Measurements of length (ABL, SBL) and width (TW) follow those of Ball (1972) and Kavanaugh (1979): ABL (apparent body length), measured from apex of labrum to apex of the abdomen; SBL (standardized body length), equals the sum of the lengths of the head (measured from apex of clypeus to a point on midline at level of the posterior edge of compound eyes), PL (pronotal length ), measured from apical to basal margin along midline, and LE (elytron length), measured from apex of scutellum to apex of the longer elytron; and TW (total width), measured across both elytra at their widest point with suture closed.”

“Habitus and attribute images of the adult beetles portray most of the character states referred to in the key provided. Male and female genitalic presentations are standard for descriptive taxonomy of carabid beetles, and in this case are digital photo-illustrations (Erwin 2011). The images of the adult and its parts were made with a Visionary Digital^TM^ high resolution imaging system. Figure captions include an ADP number, which is a unique identification number for the specimen that was illustrated or imaged and links the specimen and associated illustrations and/or image to additional information in electronic databases at the NMNH.”

“Geographical data are presented based on all known specimens of each species available at the time of manuscript preparation. Georeferences have been determined from locality information provided on specimen labels. Latitude and longitude are reported in decimal degrees. A distribution map is provided for the species [Fig. 5]. Here, an English vernacular name is proposed, as vernacular names are becoming increasingly needed in conservation and/or agricultural and forestry applications, as well as for the Encyclopedia of Life (www.eol.org),” (Erwin and Amundson in press).

## Accounts of taxa

### Western Hemisphere genera of Pseudomorphini Newman 1842

*Guyanemorpha* Erwin, gen. n. Guyane (French Guiana)

*Manumorpha* Erwin & Geraci, 2008. Brazil, Ecuador, Guyane, Perú

*Notopseudomorpha* (Baehr, 1997). Middle and South America

*Pseudomorpha* (s. str.) Kirby, 1825. USA south to Argentina (incl. Caribbean islands)

*Samiriamorpha* Erwin & Geraci, 2008. Perú

*Tuxtlamorpha* Erwin & Geraci, 2008. México, Honduras

*Yasunimorpha* Erwin & Geraci, 2008. Ecuador

### Key to the Western Hemisphere genera of Pseudomorphini Newman 1842

**Table:** 

1	Mouthparts not visible in dorsal aspect. Preocular lobe absent	2
1’	Mouthparts visible in dorsal aspect. Preocular lobe present	3
2(1)	Dorsal surface glabrous, markedly shiny	*Notopseudomorpha* (Baehr, 1997)
2’	Dorsal surface finely setiferous, not shiny	*Samiriamorpha* Erwin & Geraci, 2008
3(1’)	Elytron with only scutellar and ombilicate setae; with elytra markedly tapered to apex	4
3’	Elytron multisetiferous; body form rather broad and subdepressed with elytra not or barely tapered to broadly round apex	5
4(3’)	Body form narrow, somewhat cylindrical	*Yasunimorpha* Erwin & Geraci, 2008
4’	Body form very broad, not cylindrical	*Guyanemorpha* gen. n.
5(3)	Dorsal surface with dense vestiture, of very long thick erect setae equal in length at least to basal 4 antennomeres, but no pubescence; body form subconvex, elytra tapered posteriorly	*Manumorpha* Erwin & Geraci, 2008
5’	Dorsal surface with sparse or no long vestiture, longer setae equal in length only to at most basal 3 antennomeres, also usually with short pubescence; body form subconvex, elytra slightly tapered posteriorly or not	6
6(5’)	Major setae of dorsal surface erect or slightly curved posteriorly	*Pseudomorpha* Kirby, 1825
6’	Major setae of elytra posteriorly directed and markedly decumbent	*Tuxtlamorpha* Erwin & Geraci, 2008

#### Pseudomorphini

Newman, 1842

http://species-id.net/wiki/Pseudomorphini

Pseudomorphini Newman, 1842: 365 (as Pseudomorphites)

##### Proposed english vernacular name.

False-form beetles.

##### Taxonomy.

Stable at the generic level.

##### Classification.

According to Ober and Maddison (2008), Pseudomorphini appears as a branch of the higher Carabidae and associated with Graphipterini and Orthogonini; according to Erwin and Geraci (2008), the adelphotaxon is the tribe Orthogonini. All three tribes are associated in some way with ants or termites. Male genitalia of pseudomorphines have a bonnet-shaped phallobase as in the lebiomorphs, yet their accompanying parameres are large and nearly symmetrical (and in some species the parameres are sparsely setiferous), as in some primitive lineages of the family. Many known lineages of Pseudomorphini have been so highly selected for life with ants (and possibly termites) that external structures do not help much in discovering more normal carabid relatives (cf. Erwin and Amundson, in press).

##### Taxonomy references.

Baehr (1992, 1997); Erwin and Amundson (in press); Erwin and Geraci (2008); Notman (1925), Ogueta (1967).

##### Larval references.

Erwin (1981); Lenko (1972); Liebherr and Kavanaugh (1985), Moore (1964, 1974, 1983).

#### Guyanemorpha

Erwin gen. n.

http://zoobank.org/66A2E5B7-0831-4E1E-8AD2-ECE683F0AD8D

http://species-id.net/wiki/Guyanemorpha

##### Type species.

*Guyanemorpha spectabilis* Erwin, sp. n.

##### Proposed english vernacular generic name.

Guyane False-form beetles

##### Adelphotaxon.

Probably *Notopseudomorpha* (Baehr, 1997) (see Erwin and Geraci 2008 for phylogeny).

##### Description.

**Figure 2–3.**
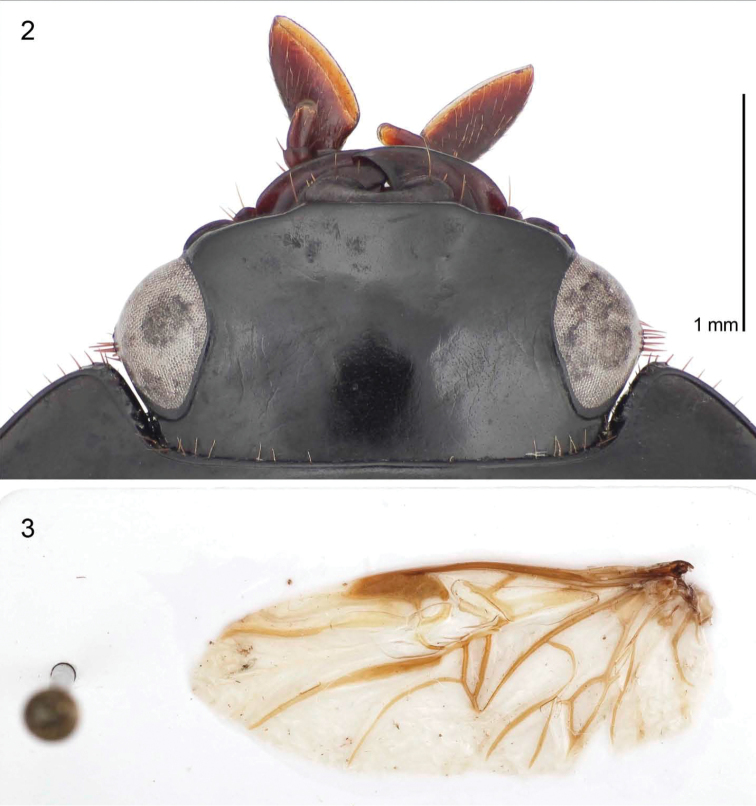
**2** *Guyanemorpha spectabilis* sp. n., female holotype, ADP132101; Risquetout, PK20, Guyane. Head, dorsal planar aspect **3** *Guyanemorpha spectabilis* sp. n., female paratype, ADP132105; Nouragues, Saut Pararé, Guyane. Left flight wing, dorsal aspect.

**Head** (Fig. 2) without supraorbital setigerous punctures, nor any accessory setae; frontal impressions absent. Labrum barely visible with anterior margin shallowly emarginate, quadrisetose; clypeus markedly wide, nearly obscured in dorsal aspect by protruding frons, with obtuse setiferous lateral corners. Eyes slightly convex; small gena with numerous stout setae. Antenna short, just reaching anterior coxa in repose; antennomeres 3-9 slightly wider than 1-3, and appearing slightly flattened. Mandible markedly flattened with a very short and acute apex; outer margin ventral of the scrobe without short stout setae. Maxillary palpi markedly short, 3-segmented, palpomeres slightly depressed, palpomere 3 truncate apically. Labial palpus with short bisetose palpomere 2; palpomere 3 markedly securiform and robust, its distal margin mostly membranous with sensory organs.

**Prothorax.** Pronotum (Fig. 1) much wider than head, transverse, with narrowly beaded margins; without a pair of setigerous punctures each side, apical, lateral and posterior margins with fringe of short stout setae; hind angles obtuse, broadly rounded. Prosternal process unisetiferous subapically, intercoxal process feebly beaded.

**Pterothorax.** Metepisternum elongate and markedly narrowed posteriorly, the outer margin about 2 times greater in length than the anterior margin, posterior margin about 0.2 times anterior margin.

**Elytra.** Elytron (Fig. 1) tapered, markedly narrower apically, width of elytra about equal to that of pronotum at widest point, apical margin truncate with evenly rounded humerus, interneurs and intervals effaced; parascutellar stria absent, scutellum hidden, parascutellar puncture present, marked; without fixed discal setae, surface glabrous. Lateral marginal (umbilical) series of 10 setae, arrayed throughout and widely spaced; lateral margin with fringe of short stout setae.

**Hind wings.** Macropterous. Venation (Fig. 3).

**Legs.** Short and depressed, femur posteriorly channeled for reception of tibia in repose; antennal comb notch very shallow; tibial spurs normal; anterior tarsi of male with tarsomeres 2–4 dilated slightly, ventrally each with two laterally placed rows of adhesive articulo-setae.

**Abdomen.** Abdominal sterna III–VII with patches of short setae and each of IV–VII with a single row of erect ambulatory setae numbering 2 to 8 setae; V and VI in male each with dense row of yellowish robust setae separated medially.

**Figure 4.**
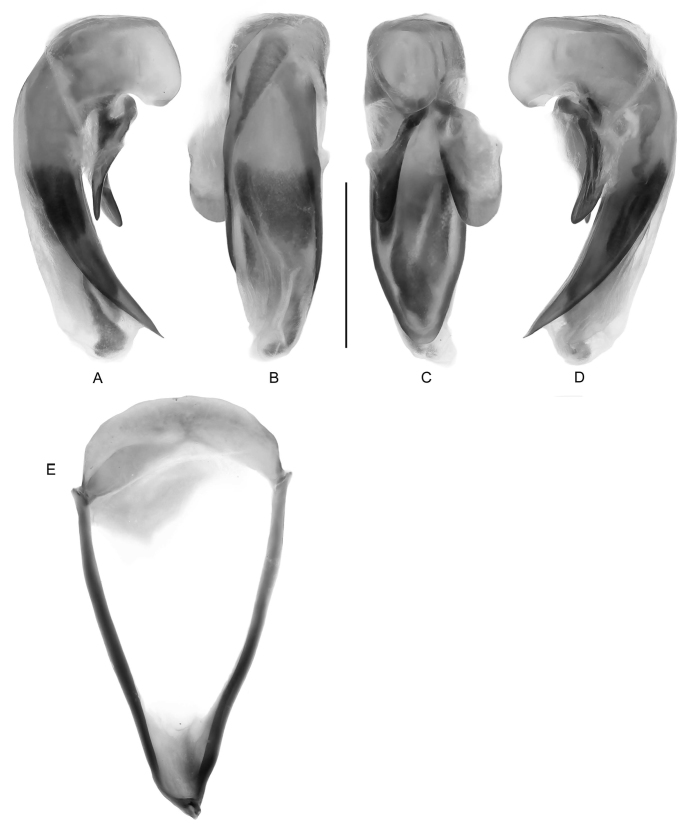
*Guyanemorpha spectabilis* sp. n., male paratype, ADP132103; Risquetout, PK20, Guyane. Male genitalia, median lobe and parameres labeled as in repose in male **A** ventral aspect **B** right lateral aspect **C** left lateral aspect **D** dorsal aspect.

**Male genitalia** (Fig. 4). Phallobase hooded with small orifice, dorsum not crested; phalloshaft arched throughout its length, diameter sub-rounded to somewhat depressed dorso-ventrally; phalloapex produced, sharp, rounded, markedly depressed dorso-ventrally; endophallus orifice elongate, endophallus with dense patches of microtrichia. Parameres (C) moderately short compared to those of genus *Pseudomorpha*, nearly equal in length, left slightly longer and much broader than right, each apically glabrous. Ring sclerite (E) normal for family.

**Female ovipositor and genital tract** (Fig. 1). Gonocoxite 2 falcate, base about as long as blade, latter relatively short, pointed distally; margins without ensiform setae; with short preapical nematiform seta.

#### Guyanemorpha spectabilis

Erwin sp. n.

http://zoobank.org/B86F564C-10FD-43CD-B257-DDE8425CF0D7

http://species-id.net/wiki/Guyanemorpha_spectabilis

Figures 1 –5

##### Holotype.

**Guyane (French Guiana):** Risquetout, PK20, 4.916°N, 52.516°W, 12m, 13 December 2010 (SEAG) (NMNH, held in trust, see below: ADP132101, female). Paratypes are listed below under other specimens examined.

##### Derivation of specific epithet.

The epithet “*spectabilis*” is a Latin adjective describing the very large and colorful beetle species.

##### Proposed english vernacular name.

Spectacular Guyane False-form beetle.

##### Diagnosis.

With the attributes of the genus as described above and color black and rufous with elytral spots (Fig. 1), color tone of head and pronotum uniform black; form broad and stout with tapered elytra; head with preapical lobe prominent but hidden in dorsal aspect beneath the frons, about 2/3 the length of the anterior margin of eye; pronotum (Fig. 1) coequal at base to elytra across humeri; elytron markedly tapered from humerus to narrower truncated apex and without interneurs or intervals, surface glabrous except parascutellar seta and 8 ombilicate setae near lateral margin.

##### Description.

(Figs 1, 2, 3, 4; Appendix 3). Size: Very large for a Western Hemisphere species, ABL = 13.18 to 13.51 mm, SBL = 11.05 to 12.18 mm, TW = 6.36 to 6.86 mm. Preocular lobe-eye ratio (L/L): 0.49 to 0.54. Pronotum ratio (L/W): 1.99 to 2.16. Pronotum ratio (W/L): 2.20 to 2.29. As described for genus above and the diagnosis.

##### Dispersal potential.

These beetles are macropterous and have been recorded from flight intercept traps (FITs), hence fully capable of flight; they are likely swift and agile runners as other species in the Tribe. Accordingly, this species may be expected to be more broadly distributed across a wider geographical range than current records indicate.

##### Way of life.

Adults of other pseudomorphines in the Western Hemisphere are found in ant nests and the surrounding vicinity and possibly in termite nests (Ogueta 1967); female adults of species of *Pseudomorpha* are ovoviviparous (Liebherr and Kavanaugh 1985); *Pseudomorpha* and *Notopseudomorpha* larvae are ant nest inquilines (Erwin 1981, Lenko 1972). Members of *Guyanemorpha spectabilis* occur at lowland rainforest sites and most likely live with ants. They have been found in July and December.

##### Other specimens examined.

**Guyane:** Réserve Trésor, 4.610°N, 52.279°W, 225m, 11 July 2009 (S. Brule) (BMNH, ADP124772, male paratype); Risquetout, PK20, 4.916°N, 52.516°W, 12m, 13 December 2010 (J.L. Giuglaris) (MNHP: ADP132103); Nouragues, Saut Pararé, 4.02°N, 52.41°W, 51m, 14 July 2010 (SEAG) (MNHP, ADP132105, female paratype).

##### Geographic distribution.

**Figure 5.**
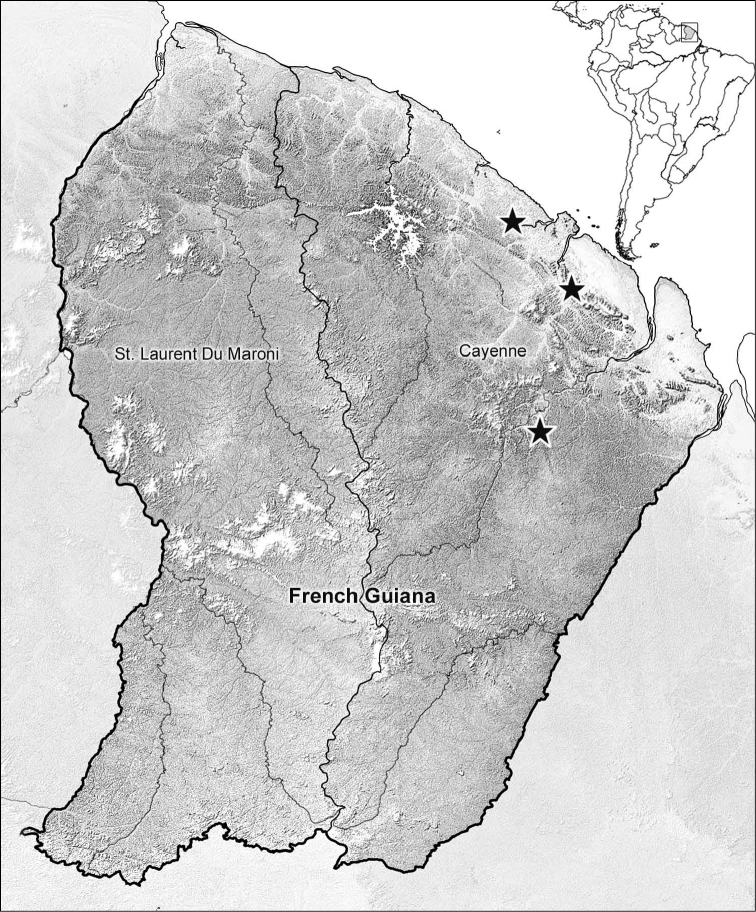
Distribution dot map for known localities of *Guyanemorpha spectabilis* sp. n.

(Fig. 5). This species is currently known only from Guyane.

##### Note.

The holotype is currently held in trust at the National Museum of Natural History, Smithsonian Institution, Washington, DC until the planned Natural History Museum of Guyane is established, and at that time the specimen will be transferred there. See details of SEAG carabid collections in Erwin et al. 2012.

## Concluding statement

Adults of *Guyanemorpha* share attributes with those of both *Notopseudomorpha* (Baehr) and *Yasunimorpha* Erwin & Geraci. This surprising large and colorful pseudomorphine came as a shock to the author, as all other species of the Tribe in the Western Hemisphere are quite dull fulvous, rufous, or black with no, or little, color contrast on the dorsal surface. Since little in the way of life information is available for the four specimens reported herein except seasonality of adult activity and lowland habitation, only speculation based on fogging samples in Perú and Ecuador can add much to this conclusion. “The pseudomorphines are a very interesting evolutionary off-shoot of the normal carabid morphotype in both form and function and are only just now beginning to be understood in North America. The fact that species of related genera in South America are living with arboreal ants will make learning about them far more difficult. Insecticidal fogging gets adults of these species, but only tearing apart arboreal *Azteca* ant nests while suspended in a tree will produce their larvae, and that is not for carabidologists faint of heart.” Erwin and Amundson (in press).

## Supplementary Material

XML Treatment for Pseudomorphini

XML Treatment for Guyanemorpha

XML Treatment for Guyanemorpha spectabilis

## Appendix 1

Institutions and personnel who loaned specimens for this revision.

**BMNH** Maxwell V. L. Barclay, Curator and Collection Manager, Entomology: Coleoptera & Hemiptera, Department of Life Sciences, Natural History Museum, London SW7 5BD

**SEAG** S. Brule, J.L. Giuglaris, The Entomological Society Antilles-Guyane, stephanebrule973@hotmail.fr

## Appendix 2

Errata: In Erwin and Geraci (2008), Figure captions 13 and 14 are reversed. Fig. 13 is *Notopseudomorpha* sp., Costa Rica, and fig. 14 is *Pseudomorpha excrucians* Kirby.

## Appendix 3

**Table 1.** Measurements and ratios for *Guyanemorpha spectabilis* sp. n., all measurements are in millimeters.

**Total length (sbl)**					
Males			Females		
N	Range	Mean	N	Range	Mean
2	12.079–12.135	12.107	2	11.053–12.185	11.844
**Maximum width (tw)**					
Males			Females		
N	Range	Mean	N	Range	Mean
2	6.356–6.378	6.367	2	6.41–6.864	6.637
**W of head/w of left elytron**					
Males			Females		
N	Range	Mean	N	Range	Mean
2	1.041–1.069	1.055	2	0.980–0.991	0.986
**Pronotum: width (at widest part)/length**					
Males			Females		
N	Range	Mean	N	Range	Mean
2	2.193–2.220	2.207	2	2.253–2.327	2.29
**Length of pronotum / length of head**					
Males			Females		
N	Range	Mean	N	Range	Mean
2	1.959–2.027	1.993	2	2.040–2.285	2.163
**Apparent body length (abl)**					
Males			Females		
N	Range	Mean	N	Range	Mean
2	13.510–14.480	13.995	2	13.180–14.180	13.68
**Pre-ocular length / eye length**					
Males			Females		
N	Range	Mean	N	Range	Mean
2	0.435–0.554	0.494	2	0.520–0.551	0.535

## References

[B1] Baehr M (1992) Revision of the Pseudomorphinae of the Australian region. 1. The previous genera *Sphallomorpha* Westwood and *Silphomorpha* Westwood. Taxonomy, phylogeny, zoogeography. (Insecta, Coleoptera, Carabidae). Spixiana, Supplement 18: 1-439.

[B2] Baehr M (1997) Revision of the Pseudomorphinae of the Australian region. 2. The genera *Pseudomorpha* Kirby, *Adelotopus* Hope, *Cainogenion* Notman, *Paussotropus* Waterhouse, and *Cryptocephalomorpha* Ritsema. Taxonomy, phylogeny, zoogeography. (Insecta, Coleoptera, Carabidae). Spixiana, Supplement 23: 1-508.

[B3] Ball GE (1959) A taxonomic study of the North American Licinini with notes on the Old World species of the Genus *Diplocheila* Brullé (Coleoptera). Memoirs of the American Entomological Society 16: iv + 1–258.

[B4] Ball GE (1972) Classification of the species of *Harpalus* subgenus *Glanodes* Casey (Carabidae: Coleoptera). The Coleopterists Bulletin 26: 179-204.

[B5] Erwin TL (1981) A synopsis of the immature stages of Pseudomorphini (Coleoptera: Carabidae) with notes on tribal affinities and behavior in relation to life with ants. The Coleopterists Bulletin 35(1): 53-68.

[B6] Erwin TL (2000) A new genus and species of Lachnophorini and two new species of Lebiini from Costa Rica (Coleoptera: Carabidae). The Coleopterists Bulletin 54(3): 279-283. doi: 10.1649/0010-065X(2000)054[0279:ANGASO]2.0.CO;2

[B7] Erwin TL (2004) The Beetle Family Carabidae of Costa Rica and Panamá: Descriptions of four new genera and six new species with notes on their way of life (Insecta: Coleoptera). Zootaxa 537: 1-18.

[B21] Erwin TL (2011) Rainforest understory beetles of the Neotropics, Mizotrechus Bates 1872, a generic synopsis with descriptions of new species from Central America and northern South America (Coleoptera, Carabidae, Perigonini). ZooKeys 145: 79–128. doi: 10.3897/zookeys.145.2274 PMC3267457 22287885

[B8] Erwin TL Geraci CJ (2008) New genera of Western Hemisphere Pseudomorphini (Insecta: Coleoptera, Carabidae). In: Penev L Erwin T Assmann T (Eds) Back to the Roots and back to the future: towards a new synthesis between taxonomic, ecological, and biogeographical approaches in carabidology. Proceedings of the XIII European Carabidologists Meeting, Blagoevgrad, August 20–24, 2007 Pensoft Publishers, Sofia, Bulgaria, 77-100.

[B9] Erwin TL Johnson PJ (2000) Naming species, a new paradigm for crisis management in taxonomy: Rapid journal validation of scientific names enhanced with more complete descriptions on the internet. The Coleopterists Bulletin 54(3): 269-278. doi: 10.1649/0010-065X(2000)054[0269:NSANPF]2.0.CO;2

[B10] Erwin TL Micheli C Hevel G (2012) Neotropical beetles of Guyane: Genera of the family Carabidae (Coleoptera) with notes on species richness currently known from the literature and recent collections. Le Coléoptériste 5: 1-88.

[B11] Erwin TL Kavanaugh DH (1981) Systematics and zoogeography of *Bembidion* Latreille: I. The *carlhi* and *erasum* groups of western North America (Coleoptera: Carabidae, Bembidiini). Entomologica Scandinavica, Supplement 15: 33-72.

[B12] Kavanaugh DH (1979) Studies on the Nebriini (Coleoptera: Carabidae), III. New Nearctic *Nebria* species and subspecies, nomenclatural notes, and lectotype designations. Proceedings of the California Academy of Sciences 42: 87-133.

[B22] Kavanaugh DH Erwin TL (1991) The tribe Cicindini Bänninger (Coleoptera: Carabidae): Comparative morphology, natural history, and reclassification. Proceeding of the Entomological Society of Washington 93(2): 356–389.

[B13] Kirby W (1825) A description of some insects which appear to exemplify Mr. William S. MacLeay’s doctrine of affinity and analogy. Transactions of the Linnean Society of London 14: 93-110. doi: 10.1111/j.1095-8339.1823.tb00081.x

[B14] Lenko K (1972) *Pseudomorpha laevissma*, um carabideo mirmecofilo (Coleoptera: Carabidae). Studia Entomologica 15: 439-444.

[B15] Liebherr JK Kavanaugh DH (1985) Ovoviviparity in carabid beetles of the genus *Pseudomorpha* (Insecta: Coleoptera). Journal of Natural History 19: 1079-1086. doi: 10.1080/00222938500770681

[B16] Moore BP (1964) Australian larval Carabidae of the subfamilies Broscinae, Psydrinase, and Pseudomorphinae (Coleoptera). Pacific Insects 6: 242-246.

[B17] Moore BP (1974) The larval habits of two species of *Sphallomorpha* Westwood (Coleoptera: Carabidae: Pseudomorphinae). Journal of the Australian Entomological Society 13: 179-183. doi: 10.1111/j.1440-6055.1974.tb02171.x

[B18] Moore BP (1983) A guide to the beetles of south-eastern Australia, fasc. 5: 69–84. Australian Entomological Press, Greenwich.

[B19] Newman E (1842) List of Insects collected at Port Philipp, South Australia, by Edmund Thomas Higgins, Esq. Entomologist 23: 361-369.

[B23] Notman H (1925) A review of the beetle family Pseudomorphidae, and a suggestion for a rearrangement of the Adephaga, with descriptions of a new genus and new species. Proceedings of the United States National Museum 67(14): 1–34.

[B24] Ober KA Maddison DR (2008) Phylogenetic relationships of tribes within Harpalinae (Coleoptera: Carabidae) as inferred from 28S ribosomal DNA and the wingless gene. Journal of Insect Science 8(63): 1–32. 10.1673/031.008.6301 PMC3127421 20302528

[B20] Ogueta E (1967) Las especies argentinas de la subfamilia Pseudomorphinae G. Horn, 1881. Acta Zoológica Lilloana 23: 217-232.

